# High Level 3D Structure Extraction from a Single Image Using a CNN-Based Approach

**DOI:** 10.3390/s19030563

**Published:** 2019-01-29

**Authors:** J. A. de Jesús Osuna-Coutiño, Jose Martinez-Carranza

**Affiliations:** 1 Department of Computer Science, Instituto Nacional de Astrofísica, Óptica y Electrónica, 72840 Puebla, Mexico; carranza@inaoep.mx; 2 Department of Computer Science, University of Bristol, Bristol BS8 1TH, UK

**Keywords:** high level 3D structure extraction, depth data analysis, CNN, single image, 3D vision

## Abstract

High-Level Structure (HLS) extraction in a set of images consists of recognizing 3D elements with useful information to the user or application. There are several approaches to HLS extraction. However, most of these approaches are based on processing two or more images captured from different camera views or on processing 3D data in the form of point clouds extracted from the camera images. In contrast and motivated by the extensive work developed for the problem of depth estimation in a single image, where parallax constraints are not required, in this work, we propose a novel methodology towards HLS extraction from a single image with promising results. For that, our method has four steps. First, we use a CNN to predict the depth for a single image. Second, we propose a region-wise analysis to refine depth estimates. Third, we introduce a graph analysis to segment the depth in semantic orientations aiming at identifying potential HLS. Finally, the depth sections are provided to a new CNN architecture that predicts HLS in the shape of cubes and rectangular parallelepipeds.

## 1. Introduction

In computer vision, High-Level Structure (HLS) extraction consists of recognizing 3D elements from a set of images. There are several HLS that can be extracted (lines, planes and polyhedrons), and several approaches for HLS extraction have been proposed. In general, the use of HLS provides rich scene information since in man-made scenes (urbanized environments), there exist abundant HLS. In addition, HLS reduces computational processing by covering large areas with a few parameters. Due to these characteristics (rich scene information and computational processing reduction), several tasks use HLS in order to perform improvements, for example: robotics [1], augmented reality [2], navigation [3], 3D reconstruction [4] and Simultaneous Localization and Mapping (SLAM) [5].

There exist several approaches for HLS extraction: the first analyses two or more images captured from different camera views [6,7]. This approach has high performance under image sequences (collections of images related by time, such as frames in a movie or magnetic resonance imaging). Unfortunately, sufficient parallax is necessary, i.e., some difference between camera views to reach accurate results. Other approach associates HLS with a 3D point cloud [8,9,10]. These methods rely on fitting algorithms, typically RANSAC and some optimization techniques to fit 3D structures within 3D point clouds. Nevertheless, several thresholds and a specific setup are required in order to guarantee high performance for a specific scene. This is an important limitation because in several cases, it is difficult to set appropriate thresholds for setting up values.

The use of depth sensors is another approach to HLS extraction [11,12,13]. This approach in most case uses algorithms to build the 3D model on depth information, i.e., this uses algorithms that can complete the unobserved geometry using a prediction computed from the observed depth. Unfortunately, RGBD sensors often deliver low stability under outdoor scenarios. In addition, they are not present in personal devices (cell phones, personal assistants, personal computers, etc.). Finally, the power computation, cost and size are higher than RGB sensors.

Another approach, and which we are interested in this research, is the extraction of HLS from a single image [14]. Unlike the other trends (using two views or using 3D point clouds), this approach extracts HLS without parallax constraint and without threshold values. This is useful because in real-world applications, several data are limited to a single view from an unknown scene, for example historical images, Internet images, personal pictures, holiday photos and so on. Therefore, in the current work, HLS from a single image represents a promising solution with high performance. However, there are several challenges because there is insufficient information recorded in an image. In recent work [15,16], important progress in 3D structure interpretation has been made. This was achieved via learning algorithms that learn the relationship between visual appearance and scene structure. Motivated by the results of such techniques and the potential benefits that single-image perception provide (HLS extraction without parallax constraints and without threshold values), this work focuses on HLS extraction from a single image. We believe this is a very interesting task since, despite the considerable challenges involved, some kinds of single-image structure interpretation do indeed seem to be possible.

Following, in Section 2, we present the related works that determine the location of the research; Section 3 contains our proposed method; Section 4 describes the experiments designed to evaluate the feasibility of the proposed method and the results achieved; finally, the conclusions and future work are indicated in the last section.

## 2. Related Work

In recent work, important progress on HLS extraction from a single image has been made. One popular trend uses an approach without depth information with techniques such as geometric recognition, vanishing points and learning algorithms, among others. Another approach is HLS extraction with depth estimation from a single image. In most cases, this approach uses learning algorithms that learn the relationship between visual appearance and depth information.

### 2.1. HLS Extraction without Depth Estimation

The HLS extraction without depth estimation from a single image provides a direct formulation. The approach proposed by [14,17] interprets the geometric context from a single image using a learning algorithm. This geometric context is assigned to one of three main classes, ground, sky and vertical, of which the latter is further subdivided into left, right, forward, porous and solid. Although this approach is not explicitly aimed at HLS detection, it has an understanding of the general structure of scenes, as the image is partitioned into planar structures (ground, left, right, forward) and non-planar structures (sky, solid, porous). The classification of this approach is achieved using a large variety of features, including colour (summary statistics and histograms), filter bank responses to represent texture, image location, line intersections, shape information and vanishing point. These cues are used in the various steps of classification, using decision trees and logistic regression to select the geometric context.

The approach presented by [18,19,20] shows a methodology to extract dominant planar structures by analysing the pattern of the lines and vanishing points of an image. The method is based on the assumption that there are three orthogonal directions presented in the scene. In addition, to find rectangular surfaces from them, two pairs of lines, corresponding to two different vanishing points, are used to localize planar structures. This approach has shown promising results, in both indoor and outdoor scenes, and the authors mentioned that it would be useful for robot navigation and Augmented Reality (AR) applications. Unfortunately, it is limited to scenes with planar structures and perpendicular orientation. Another approach, presented by [16,21] is the planar structures’ extraction and their orientation using a learning algorithm. For that, it selects a subset of salient points of the image, the features of which will be extracted. In this approach, two features are obtained: The first is a gradient orientation histograms, which consist of histograms of edge orientation. Second is the colour using RGB histograms, created by histograms from the red, green and blue channels. To reduce the dimensionality of the distribution of features in an image region, bag of words is used. Finally, a learning algorithm is used to take into consideration the relations between planar surfaces and their features (gradient orientation and colour). These approaches have shown promising results in outdoor scenes; in most of the cases, they are limited to plane sections’ recognition without providing polyhedral structures’ extraction.

The method presented by [22] proposed PoseCNN, a new convolutional neuronal network for the estimation of objects postures. PoseCNN is trained to perform three tasks: semantic labelling, 3D translation and 3D object rotation. The network contains two stages. The first stage consists of 13 convolutional layers and four max pooling layers, which extract feature maps with different resolutions from the input image. The second stage consists of an embedding step that embeds the high-dimensional feature maps generated by the first stage. Then, the network performs three different tasks that lead to the pose estimation, i.e., semantic labelling, 3D translation estimation and 3D rotation. The method presented by [23] addresses the problem of 3D structure reconstruction from a single image, presenting 3D reconstruction in a point cloud. This approach uses deep neural networks. The 3D reconstruction network consists of two steps. First, the input image is provided to the “encoder”; this step accommodates the input information in Step 2. Step 2 provides 3D information in an N×3M matrix, where 3M are the coordinates (*x*, *y*, *z*) and *N* are the points that make up the 3D object. Step 2 is composed of two branches: one branch provides the 3D description of complex structures, and the second provides the 3D description of smooth surfaces. However, the previously-presented methods are limited to specific 3D shapes’ extraction, i.e., they only extract specific objects. Furthermore, these methods do not present polyhedral structures’ extraction on buildings or outdoor scenes.

### 2.2. HLS Extraction with Depth Estimation

There are several methods for depth estimation for a single image [24,25,26,27]. In most cases, the depth methods for a single image estimate depth using learning algorithms. One popular approach to HLS extraction uses this depth estimation as the keystone to HLS extraction. The work in [28] introduced a methodology for estimating the ground plane structure and the 3D location of the landmarks from a robot using a single image. This work uses a supervised learning algorithm (MRF) to find the relation between image characteristics (texture and gradient) and its depth information. This method divides the original image into regions of similar textures using superpixels to feedback the depth map and locate the ground plane. The method presented by [15] estimates depth maps for single images of outdoor scenes for creating 3D models with plane sections. For that, this method segments the image into superpixels and computes three features (texture variations, texture gradients and colour). These features allow them to estimate both relative and absolute depth, as well as local orientation. Furthermore, for each superpixel and respective features, it uses an MRF to infer a set of “plane parameters” that capture both the 3D location and 3D orientation. However, it limits 3D models with plane sections without providing information on polyhedral structures. The work in [29] proposed a method to estimate a ground plane structure and its depth information from a single static image. This methodology works in two steps. The first step estimates superpixel sections’ depth using a gradient boosting regression to take into consideration visual features’ relation (texture and gradient) with depth in the scene. In the second step, a RANSAC-based plane estimator uses the superpixels’ depth information to fit with the planes in the scene.

The deep neural network is another alternative to 3D structures’ extraction using depth estimation. The work in [30] proposed 3D ShapeNets, a deep learning model to represent geometric 3D shapes. This work, given a depth map, converts it into a volumetric representation. The volumetric representation is processed by 3D ShapeNets to identify the observed shape, the free space and the occluded space. The method presented by [31] proposes a network for deep volumetric shape learning. Given a collection of shapes of various objects and their different poses, the network learns the distributions of shapes of various classes by predicting the missing sections. The network has two stages: The first provides a condensed representation. In the second stage, the network reconstructs the 3D shape using deconvolutional layers. The wok in [32] developed a 3D descriptor method to identify volumetric shapes. This work developed the design of adversarial networks that jointly train a set of a Convolution Neural Network (CNN), a recurrent neural network and an adversarial discriminator. The generator network produces 3D shape features that encourage the clustering of samples with a correct label, whereas the discriminator network discourages the clustering by assigning them misleading adversarial class labels.

Several works have demonstrated that depth estimation is highly useful for HLS extraction. Although these methods have shown promising results on 3D shapes’ extraction, in most cases, they are limited to specific objects. Furthermore, these methods do not present polyhedral structures’ extraction on buildings or outdoor scenes [31,32,33]. On the other hand, the approaches that have promising results in outdoor scenes, in most cases, are those limited to plane sections’ extraction without providing polyhedral structures’ extraction [15,29]. In this work, we propose a new HLS extraction method that aims at polyhedral structures’ extraction on outdoor scenes from a single image. For that, our method has three steps. First, we propose a depth analysis to remove uncertain depth sections, and we segment depth sections with similar orientations. Second, we introduce a graph analysis to locate depth surface sets using the depth sections with a similar orientation. Finally, the depth surface sets are provided to a new CNN architecture, which predicts 3D polyhedral structures (cubes and rectangular parallelepipeds).

## 3. The Proposed Method

**Figure 1:**
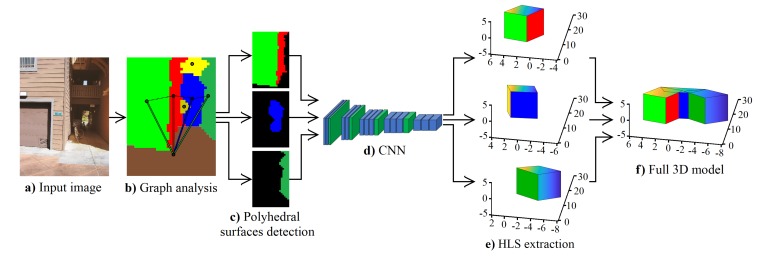
Block diagram of the proposed method. HLS, High-Level Structure.

Although learning algorithms can predict a depth map, this depth map presents several challenges to HLS extraction such as low sharpness of depth information, erroneous depth in different image sections, etc. For that, we propose a new method for HLS extraction from a single image. Our method has four steps: the use of a CNN to predict the depth, a depth analysis to remove uncertain depth sections, a graph analysis to segment the depth and a new CNN architecture that predicts HLS. Figure 1 shows the block diagram of our proposed method.

### 3.1. Depth Analysis

In this subsection, we present the proposed depth analysis to remove uncertain depth. The depth analysis elaborates, removes and replaces depth sections. Furthermore, the depth sections are labelled with a semantic orientation. For that, the depth analysis uses a decision tree and a probability technique.

#### 3.1.1. Depth Sections

**Figure 2:**
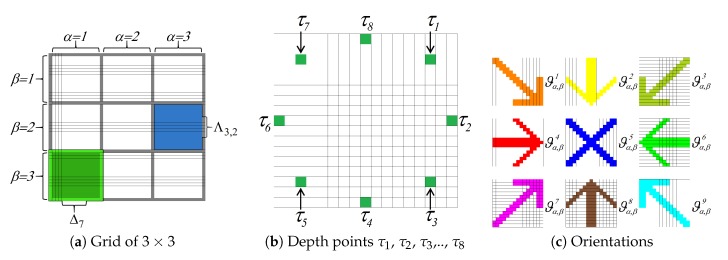
Depth analysis. Each image shows a step of the depth analysis.

We analyse the depth in sections, i.e., we do not use the depth points as independent elements; we analyse the behaviour of the depths using depth sets. For that, we use the depth images predicted by CNN [26]. Element $D_{\varepsilon }$ denotes a depth image. We divide the image $D_{\varepsilon }$ into a grid Δ. For that, the grid Δ consists of sections $\Delta_{w}$. Section $\Delta_{w}$ is a finite set of pixels $\Delta_{w}=\left\{x_{1},\ldots ,x_{n}\right\}$, $\Delta_{w}\in \Delta$, where *n* is the number of pixels in a section and $\Delta_{w}$⇔*n* is an odd number. Each section $\Delta_{w}$ has a patch $\Lambda_{\alpha ,\beta }$. Patch $\Lambda_{\alpha ,\beta }$ is a finite set of pixels $\Lambda_{\alpha ,\beta }=\left\{x_{1},\ldots ,x_{u}\right\}$, $\Lambda_{\alpha ,\beta }\in \Delta$, where, *u* is the number of pixels in a patch and $\Lambda_{\alpha ,\beta }$⇔*u* is an odd number; where *w* denotes the $w^{\mathrm{th}}$ section in grid Δ, α is the abscissa from grid Δ and β is the ordinate from grid Δ. Figure 2a shows a grid example Δ of 3×3.

#### 3.1.2. Semantic Orientation

We consider different analysis in the depth section to obtain semantic orientation. Figure 2c shows an image with the nine orientations. We use the ID3decision tree algorithm [34] to decide which analysis to use. The analyses selected were the key points analysis $\chi_{\alpha ,\beta }^{i}$ and section analysis $\gamma_{\alpha ,\beta }^{i}$. Key points analysis $\chi_{\alpha ,\beta }^{i}$ uses eight depth points. For that, the depth image $D_{\varepsilon }$ is divided into patches $\Lambda_{\alpha ,\beta }$ as in Figure 2a. In the patch $\Lambda_{\alpha ,\beta }$, we obtain the depth points $\tau_{1}$, $\tau_{2}$, $\tau_{3}$, …, $\tau_{8}$. Figure 2b shows the depth points $\tau_{1}$, $\tau_{2}$, $\tau_{3}$, …, $\tau_{8}$ for a patch $\Lambda_{\alpha ,\beta }$ of 17 × 17 pixels. To analyse the behaviour of the depth points $\tau_{i}$, we propose Equations (1) and (2).

(1) $$\begin{array}{c} \chi_{\alpha ,\beta }^{1}=\left\{\begin{array}{rr} 1 & \mathrm{if}\;\tau_{1}>\tau_{7},\tau_{2}>\tau_{6},\tau_{3}>\tau_{5},\\ 2 & \mathrm{if}\;\tau_{1}<\tau_{7},\tau_{2}<\tau_{6},\tau_{3}<\tau_{5},\\ 3 & \mathrm{if}\;\tau_{1}=\tau_{7},\tau_{2}=\tau_{6},\tau_{3}=\tau_{5},\\ 0 & otherwise, \end{array}\right. \end{array}$$

(2) $$\begin{array}{c} \chi_{\alpha ,\beta }^{2}=\left\{\begin{array}{rr} 1 & \mathrm{if}\;\tau_{5}>\tau_{7},\tau_{4}>\tau_{8},\tau_{3}>\tau_{1},\\ 2 & \mathrm{if}\;\tau_{5}<\tau_{7},\tau_{4}<\tau_{8},\tau_{3}<\tau_{1},\\ 3 & \mathrm{if}\;\tau_{5}=\tau_{7},\tau_{4}=\tau_{8},\tau_{3}=\tau_{1},\\ 0 & otherwise, \end{array}\right. \end{array}$$

The section analysis $\gamma_{\alpha ,\beta }^{i}$ divides the depth section into two sessions. For that, the depth image $D_{\varepsilon }$ is divided into patches $\Lambda_{\alpha ,\beta }$, as in Figure 2a. Equations (3) and (4) are used to compute the section analysis $\gamma_{\alpha ,\beta }^{i}$. Equation (3) divides the depth section into two horizontal sections, and they are subtracted. Equation (4) divides the depth section into two vertical sections, and they are subtracted; where the pixel of a depth image $D_{\varepsilon }$ is denoted by $k_{i,j}$ and *u* is the pixel number (row∖column) for the patch $\Lambda_{\alpha ,\beta }$.

(3) $$\gamma_{\alpha ,\beta }^{1}=\frac{\sum_{i=0}^{u}\;\sum_{j=0}^{u/2}\left(k_{i,j}-k_{i,j+\frac{u}{2}}\right)}{u^{2}/2}\;$$

(4) $$\gamma_{\alpha ,\beta }^{2}=\frac{\sum_{j=0}^{u}\;\sum_{i=0}^{u/2}\left(k_{i,j}-k_{i+\frac{u}{2},j}\right)}{u^{2}/2}$$

**Figure 3:**
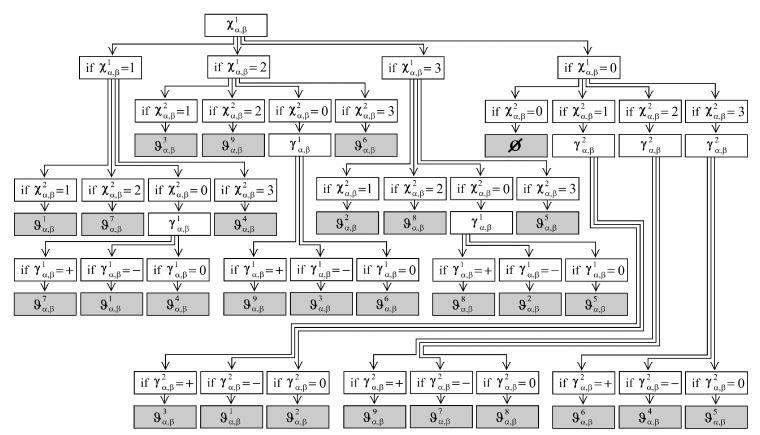
The obtained decision tree using the ID3algorithm.

To analyse the behaviour of the depth sections, we obtain their semantic orientation $\vartheta_{\alpha ,\beta }^{k}$. For that, we obtain the semantic orientation using a decision tree with the ID3 algorithm. Figure 3 shows our decision tree. Furthermore, Figure 2c shows an image with the nine orientations; where the patch $\Lambda_{\alpha ,\beta }$ is painted in orange colour if it has an orientation with a right and down view $\vartheta_{\alpha ,\beta }^{1}$. The patch $\Lambda_{\alpha ,\beta }$ is painted in yellow colour if it has an orientation with a down view $\vartheta_{\alpha ,\beta }^{2}$. The patch $\Lambda_{\alpha ,\beta }$ is painted in dark green colour if it has an orientation with a left and down view $\vartheta_{\alpha ,\beta }^{3}$. The patch $\Lambda_{\alpha ,\beta }$ is painted in red colour if it has an orientation with a right view $\vartheta_{\alpha ,\beta }^{4}$. The patch $\Lambda_{\alpha ,\beta }$ is painted in blue colour if it has an orientation with a front view $\vartheta_{\alpha ,\beta }^{5}$. The patch $\Lambda_{\alpha ,\beta }$ is painted in green colour if it has an orientation with a left view $\vartheta_{\alpha ,\beta }^{6}$. The patch $\Lambda_{\alpha ,\beta }$ is painted in purple colour if it has an orientation with a right and upward view $\vartheta_{\alpha ,\beta }^{7}$. The patch $\Lambda_{\alpha ,\beta }$ is painted in brown colour if it has an orientation with an upward view $\vartheta_{\alpha ,\beta }^{8}$. The patch $\Lambda_{\alpha ,\beta }$ is painted in sky blue colour if it has an orientation with a left and upward view $\vartheta_{\alpha ,\beta }^{9}$. Finally, if the patch $\Lambda_{\alpha ,\beta }$ does not have an orientation $\vartheta_{\alpha ,\beta }^{k}$, the patch $\Lambda_{\alpha ,\beta }$ has an uncertain orientation or null orientation ⊘. The patch $\Lambda_{\alpha ,\beta }$ is painted in black colour if it has a null orientation ⊘.

**Figure 4:**
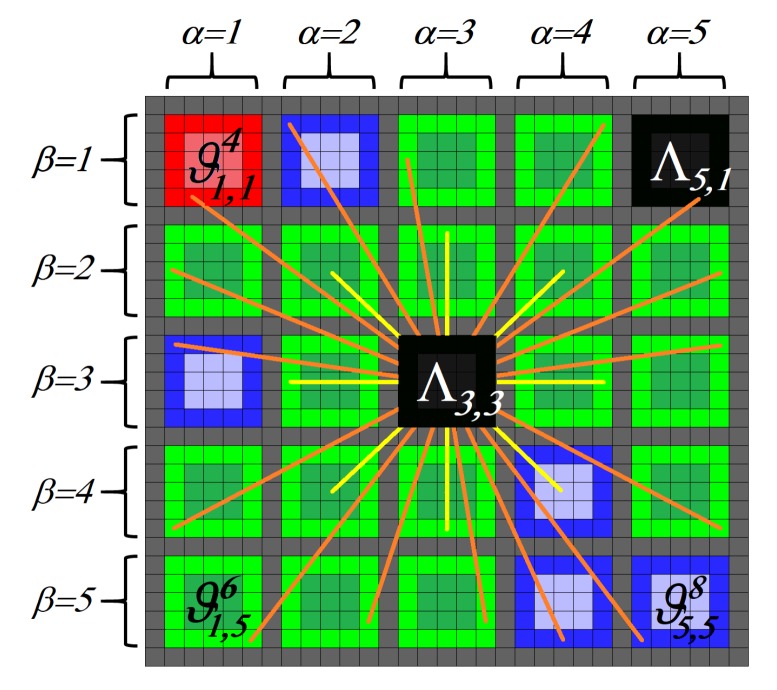
Example of patch $\Lambda_{3,3}$ with a grid Δ of 5×5.

We use the Markov chain [35] to label the patch $\Lambda_{\alpha ,\beta }$ with null orientation ⊘. The Markov chain analysis substitutes the patches $\Lambda_{\alpha ,\beta }$ with a null orientation considering the orientation neighbour. For that, we use a central patch $\Lambda_{\alpha ,\beta }$ to analyse its connection with the orientation neighbour. Figure 4 shows a central patch $\Lambda_{3,3}$ with a grid Δ of 5×5.

A stochastic matrix *P* describes a Markov chain $X_{t}$ over a finite state space with cardinality *S*. We use a right stochastic matrix that is a real square matrix, with each row summing to 1. We use $P_{i}$ to name a row of the stochastic matrix. Each of its entries $p_{i,j}$ is a nonnegative real number representing a probability. In our stochastic matrix *P*, we consider in each row the probability that the patches with null orientation have an orientation of a neighbour patch.

Considering Figure 4, our stochastic matrix *P* is a 3×3 matrix. For that, in $P_{1}$, we consider that the patches with null orientation have an orientation with a left view $\vartheta_{\alpha ,\beta }^{6}$, $P_{1}=\left\{19/25,5/25,1/25\right\}$. In $P_{2}$, we consider that the patches with null orientation have an orientation with a front view $\vartheta_{\alpha ,\beta }^{5}$, $P_{2}=\left\{17/25,7/25,1/25\right\}$. In $P_{3}$, we consider that the patches with a null orientation have an orientation with a right view $\vartheta_{\alpha ,\beta }^{4}$, $P_{3}=\left\{17/25,5/25,3/25\right\}$. In addition, we have three probability vectors, one for each orientation contemplated $V_{1}=\left\{1,0,0\right\}$, $V_{2}=\left\{0,1,0\right\}$ and $V_{3}=\left\{0,0,1\right\}$. Finally, every probability vector multiplies by the stochastic matrix *P*. If some element of the multiplication is greater than a threshold, the orientation of the element greater than the threshold enters central patch $\Lambda_{\alpha ,\beta }$. Otherwise, the resulting vector is multiplied by the stochastic matrix *P*. If the image has the same number of patches $\Lambda_{\alpha ,\beta }$ with null orientation, the threshold is reduced. The iteration ends when the image does not have null orientation. We use a threshold = 1 as the initial value.

### 3.2. Orientation Segmentation

The orientation segmentation segments the image $D_{\varepsilon }$ into sections with similar orientation patches. The orientations $\vartheta_{\alpha ,\beta }^{k}$ of patches $\Lambda_{\alpha ,\beta }$ are connected to each other; where an orientation session $\omega^{m}$ is a set of patches $\Lambda_{\alpha ,\beta }$ with similar orientation $\vartheta_{\alpha ,\beta }^{k}$ and connected to each other $\omega^{m}=\left\{\vartheta_{a,u}^{k},\ldots ,\vartheta_{e,o}^{k}\right\}$, $\omega^{m}\in \Delta$, *m* denotes the mth orientation session $\omega^{m}$, (a,e) are abscissas of the grid Δ and (u,o) are ordinates of the grid Δ.

The orientation session $\omega^{m}$ has the following properties:

(1) Patches’ connection: $\vartheta_{a,u}^{k}$ and $\vartheta_{e,o}^{k}$ are connected if there is a patch sequence with similar orientation.

(2) Disjoint region: $\omega^{i}$ and $\omega^{j}$ are disjoint regions if $\omega^{i}\cap \omega^{j}=⊘$ for all i={1,…,w}; where i≠j, *w* is the number of regions in image $D_{\varepsilon }$, ⊘ is the null set and $\left\{\omega^{i},\omega^{j}\right\}\in D_{\varepsilon }$.

(3) Segmented region: $P(\omega^{m})=TRUE$ if all pixels in $\omega^{m}$ are of a similar orientation detected by our decision tree Figure 3, where $P(\omega^{m})$ is a logical predicate defined over the points in set $\omega^{m}$ and ⊘.

### 3.3. Graph Analysis

The proposed model uses an undirected network G=(N,A), consisting of a finite set of nodes N= {1,2,3,…,n} and a set of undirected edges A= {(i,j):i,j∈N,i≠j} joining pairs of nodes in *N*. For all edges (i,j)∈A, let there be one nonnegative weight denoted by $c_{i,j}$ [36]. We consider the orientation session $\omega^{m}$ on depth image $D_{\varepsilon }$ the nodes of our graph analysis. Furthermore, the connection between two sessions $\omega^{m}$ is represented with an edge. We refer to $c_{i,j}$ as the number of connected pixels between two sessions $\omega^{m}$. Considering the edges (the different connections between two sessions $\omega^{m}$), the graph analysis locates sections with possible polyhedrons (cube, half cube and rectangular parallelepipeds) (see Figure 7).

Furthermore, each section with a polyhedron is saved in an image, removing the sky and floor. The graph analysis analyses the area of the polyhedron section and classifies the polyhedron on a cube, a half cube, a horizontal rectangular parallelepiped or a vertical rectangular parallelepiped. Finally, the depth of the polyhedron section is provided to one of the four CNN, where every CNN provides the coordinate (*x*,*y*,*z*) vertices of one HLS.

### 3.4. CNN for HLS Extraction

**Figure 5:**
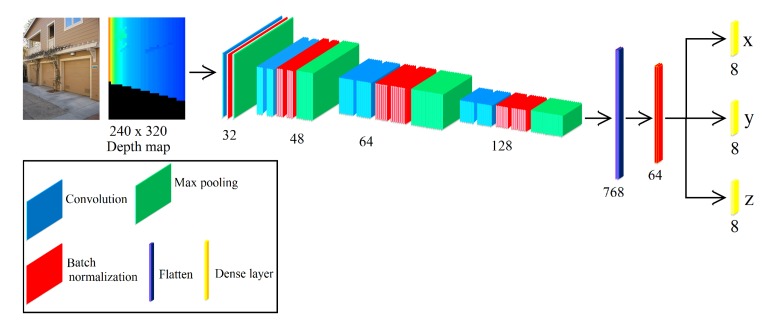
The architecture of our CNN for HLS extraction.

Our network aims to provide the coordinate (*x*,*y*,*z*) vertices of HLS from a given depth map. The depth map is obtained from the mentioned graph analysis. The proposed network accepts as input a depth map with a size of 240 × 320 pixels. In addition, our network contains two stages. The first stage consists of 7 convolutional, 7 batch normalization and 4 max pooling layers, which extract feature maps with different resolutions from the input image. This stage is the backbone of the network since the extracted features are shared in the second stage. The second stage consists of combining all the found local features of the previous convolutional layers. For that, we use a flatten layer and batch normalization. Finally, we use dense layers to obtain the coordinate (*x*,*y*,*z*) vertices of HLS. Figure 5 shows the architecture of our CNN for HLS extraction. In addition, we have four architectures of our CNN, and every CNN extracts one structure (cube, half cube, horizontal rectangular parallelepiped or vertical rectangular parallelepiped). To know what CNN has to be used, we use graph analysis (see Section 3.3). The 3D models of the HLS extraction (see Figure 9e) are obtained plotting the coordinates (*x*,*y*,*z*) in Matlab.

## 4. Discussion and Results

In this section, we present the discussion and results of the proposed HLS extraction. These discussion and results are the integration of the proposed depth analysis to remove uncertain depth sections, a new graph analysis to locate possible 3D shapes and a new CNN architecture that predicts HLS. We evaluated our approach on our simulated dataset and two datasets that provide different urbanized scenes: Make3D [15,37] and KITTI [38]. Quantitative evaluation was performed using comparisons of our depth and ground-truth depth. Furthermore, we evaluated our segmentation orientation using pixel comparisons with the ground-truth. Finally, we used the simulated dataset to evaluate our HLS extraction.

### 4.1. Depth Evaluation

We compared our depth post-processing analysis with baseline methods. We evaluated it on two popular datasets, which are available online: Make3D dataset [15,37] and the KITTI dataset [38]. To provide quantitative results, we used two measures: the root mean squared error (rms), Equation (5), and average $\log_{10}$ error ($\log_{10}$), Equation (6); where $d_{p}^{gt}$ and $d_{p}$ are the ground-truth and predicted depths respectively at the pixel indexed by *p* and *T* is the total number of pixels in all of the evaluated images.

(5) $$rms=\sqrt{\frac{1}{T}\sum_{p}\;{\left(d_{p}^{gt}-d_{p}\right)}^{2}}$$

(6) $$\log_{10}=\frac{1}{T}\sum_{p}\;\left|\log_{10}d_{p}^{gt}-\log_{10}d_{p}\right|$$

**Figure 6:**
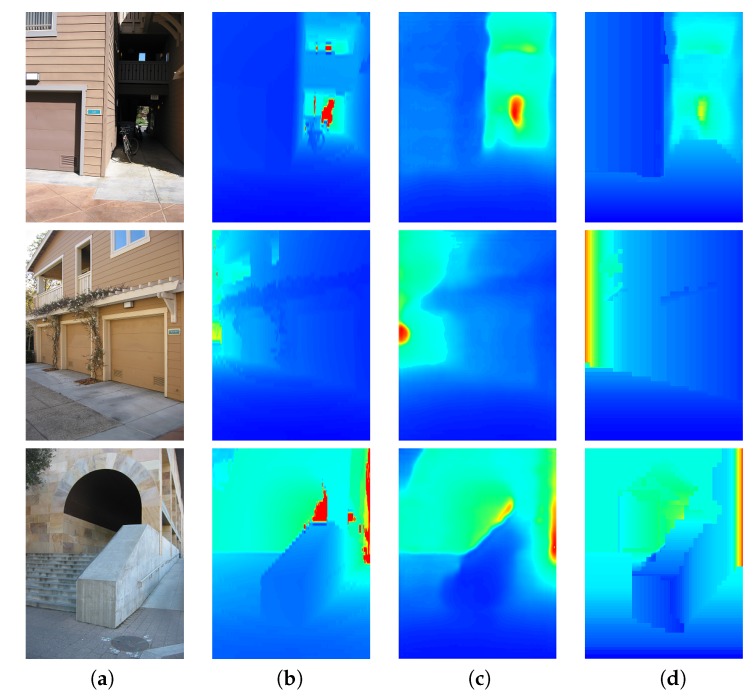
Examples of qualitative comparisons on the Make3D dataset. Colour indicates depths (red is far; blue is close). (**a**) Input image; (**b**) Ground-truth; (**c**) DCNF-FCSP [26]; (**d**) Our refined depth.

Our post-processing analysis used a predicted depth. We used the depth images predicted by DCNF-FCSP [26]. Using these depth images, we used a post-processing analysis to obtain our refined depth. Figure 6 shows the input images, ground-truth, images predicted by DCNF-FCSP [26] and our refined depth. We can see the qualitative results of the depth post-processing analysis (see Figure 6d). As can be seen, our refined depth provided an HLS depth with higher sharpness, i.e., our refined depth easily located the HLS (cubes and rectangular parallelepipeds) of the buildings. Furthermore, in Table 1, we show quantitative comparisons concerning the state-of-the-art for the Make3D [15,37] and KITTI datasets [38]. We can see that our depth post-processing analysis improved the results predicted by DCNF-FCSP [26].

### 4.2. Segmented Orientations Evaluation

In this subsection, we use the Make3D dataset [15,37] and the KITTI dataset [38] to evaluate our segmented orientations. However, to analyse our segmented orientations, we performed ground truth labelling. This ground truth consists of orientation labelling. The quantitative evaluation was performed using pixel comparisons of the obtained segmented orientations with the ground-truth. To provide quantitative results, we used three measures (recall, precision and *F-score*) based on the numbers of true positives, true negatives, false positives and false negatives. The true positives Tp count the number of pixels whose orientation label was predicted correctly w.r.t. to the ground truth. To count the number of true negatives Tn, we proceed as follows: suppose that we are interested in the orientation label *Down view*, then all those pixels corresponding to other orientations rather than down view, according to the ground truth, should have received any other predicted label except *Down view*; if that is the case, each of these pixels are counted as true negatives. The false positives Fp correspond to all those pixels whose orientation label is incorrect. Finally, false negatives Fn correspond to those pixels that should have received a specific label, but the prediction did not assign it correspondingly, for instance those portions of the image corresponding to the floor, should have received an *Upward view* label for each pixel; however, if any floor pixels did not receive such a label, then those are counted as false negatives. In terms of the measures, we carried out an analysis by each orientation label. In this sense, we used the recall to measure the proportion of pixels whose respective orientation label was predicted correctly regarding the total amount of pixels in the ground truth labelled with such an orientation label, that is in simple terms, the amount of ground truth that was predicted correctly. The precision measures the proportion of orientation labels that were predicted correctly. Finally, the *F-score* helps to summarise the performance of the predictions returned by the system. In sum, we could say that for a system with good performance, both the recall and precision should tend to one, meaning that most of the system’s predictions tend to be correct and that such predictions tend to cover most of the ground truth. If this is the case, then the *F-score* should tend to one.

(7) $$recall=\frac{Tp}{Tp+Fn}$$

(8) $$precision=\frac{Tp}{Tp+Fp}$$

(9) $$F-score=\frac{2}{\frac{1}{recall}+\frac{1}{precision}}=2\frac{recall*precision}{recall+precision}$$

Table 2 shows the results of our different segmented orientations on the Make3D [15,37] and KITTI datasets [38]. To provide quantitative results, we use three measures (recall, precision and *F-score*) on the five principal orientations (down view, right view, front view, left view and upward view), because the KITTI dataset notably only contains these orientations. Our segmented orientations have on average: a recall of 0.879758, a precision of 0.908150 and an *F-score* of 0.892359.

We deem that our results are adequate since using our orientation segmentation, we had a recall average of 0.879758, i.e., considering the ground-truth, we segmented 87.9% on average correctly. Furthermore, the results improved considering the precision, and we had a precision average of 0.908150, i.e., considering our segmented orientation, we segmented 90.8% on average correctly. In addition, the results of all our different segmented orientations had an *F-score* greater than 0.85. These results are important since the semantic orientation is an essential pre-processing step in our HLS method.

Finally, in our method, the orientation with a front view usually invades the orientations with a left and right view. This decreases the segmentation of the orientations with the left and right view (decreasing its recall). On the other hand, by invading sections that do not belong to the front view, the orientation with a front view decreases in its precision.

### 4.3. HLS Extraction Evaluation

**Figure 7:**
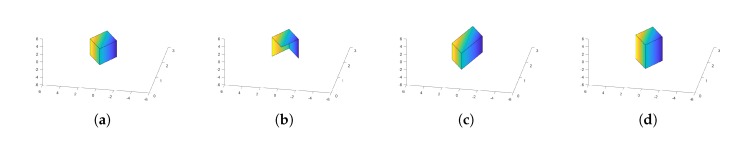
3D shapes: we used four 3D shapes (cube, half cube and rectangular parallelepipeds) to represent the HLS extraction. (**a**) Cube; (**b**) Half cube; (**c**) Horizontal rectangular parallelepiped; (**d**) Vertical rectangular parallelepiped.

We elaborated a new dataset using the Gazebo [39] simulator to evaluate the HLS extraction. The dataset consists of 152,000 images (480×640 pixels) with four 3D shapes (cube, half cube, horizontal rectangular parallelepiped and vertical rectangular parallelepiped) collected from a simulated environment. Figure 7 shows the four 3D shapes (cube, half cube, horizontal rectangle and vertical rectangle). We divided the dataset into two sets: the training set and the test set. The training set had 132,000 images, i.e., 33,000 images for every 3D shape. The test set had 20,000 images, i.e., 5000 images for every 3D shape. Therefore, there were four test sets with 5000 images and four training sets with 33,000 images. Every set (test sets or training sets) only had one 3D shape (cube, half cube, horizontal rectangular parallelepiped or vertical rectangular parallelepiped). For that, we created the 3D shape in the Gazebo simulator, and using Python, we rotated and moved the 3D shapes randomly. Finally, for every rotation and random move, we saved an RGB image, a txt with the ground-truth of depth and a txt with the ground-truth of the coordinates (*x*,*y*,*z*) of all vertices.

Furthermore, we used the training set to train the CNN and a test set to evaluate the CNN. Using depth information, the CNN provided the coordinate (*x*,*y*,*z*) vertices of a 3D shape. Finally, we extracted the HLS with the coordinates of the vertices. To evaluate the HLS extraction, we compared the coordinates of vertices predicted of the CNN with the ground-truth of the coordinates of vertices. For that, we used the root mean squared error (rms), Equation (5); where $d_{p}$ and $d_{p}^{gt}$ are the coordinates predicted by our CNN and its ground-truth respectively at coordinates indexed by *p* and *T* is the total number of coordinates in all the test set.

We use the architecture of our network to learn the coordinates of one HLS specifically, i.e., we have four CNN, and every CNN extracts one structure (cube, half cube, horizontal rectangular parallelepiped or vertical rectangular parallelepiped). To know what CNN to use, we use graph analysis (see Section 3.3). In Table 3, we show the HLS extraction evaluation with different training (the images number used to train our CNN) for every structure (cube, half cube, horizontal rectangular parallelepiped or vertical rectangular parallelepiped). As can be seen, the network gets better generalization abilities with the increase of the images number, i.e., the CNN gets better coordinate prediction if we increase the image number used to train. Furthermore, the coordinate prediction by our four CNN architectures (trained with 33,000 images) had an rsm average between 0.35 and 0.36. This shows the stability in the prediction of HLS coordinates.

**Figure 8:**
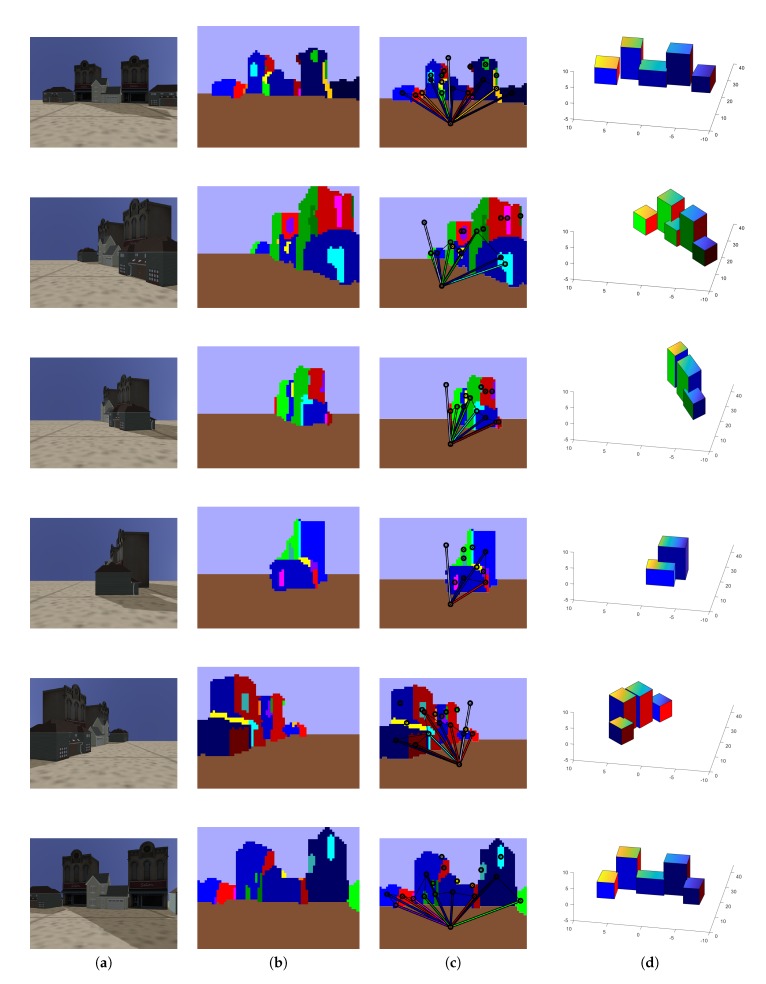
Our HLS extraction method on our simulated images. (**a**) Input image; (**b**) Orientation segmentation; (**c**) Graph analysis; (**d**) HLS extraction.

**Figure 9:**
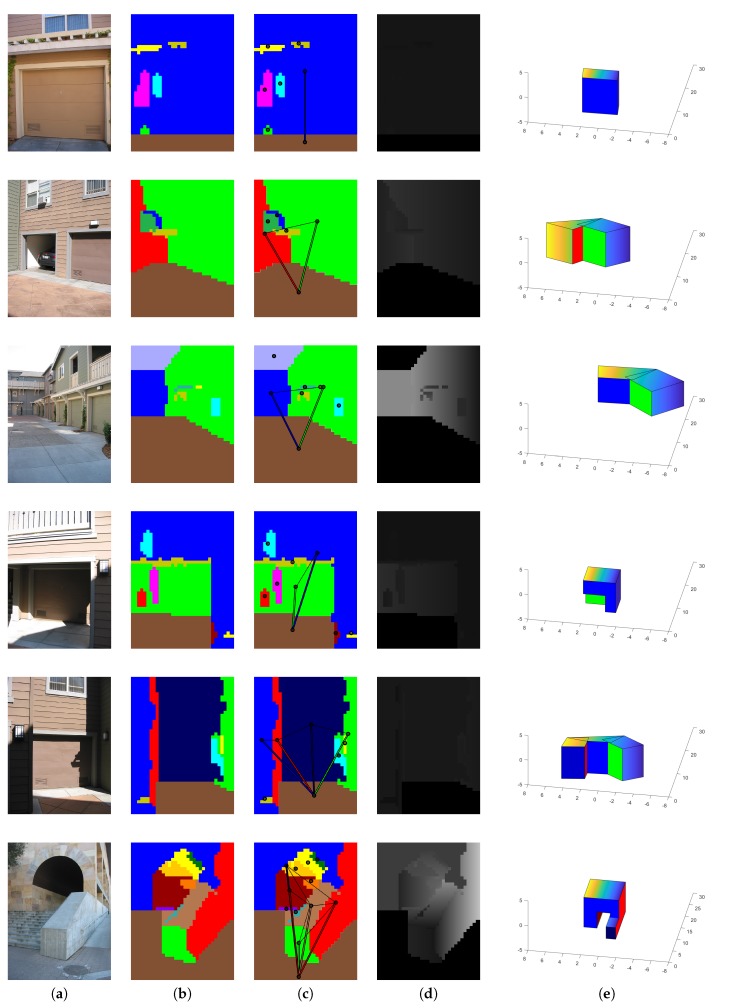
Our HLS extraction method on the Make3D dataset. (**a**) Input image; (**b**) Orientation segmentation; (**c**) Graph analysis; (**d**) Our depth; (**e**) HLS extraction.

**Figure 10:**
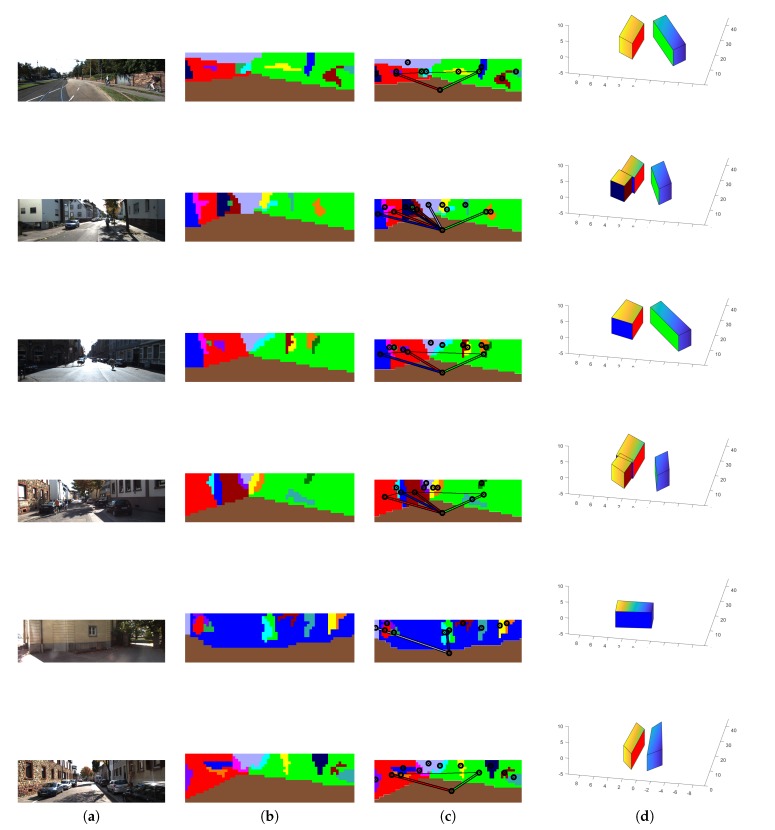
Our HLS extraction method on the KITTI dataset. (**a**) Input image; (**b**) Orientation segmentation; (**c**) Graph analysis; (**d**) HLS extraction.

In addition, to analyse the robustness of our HLS extraction method with our four CNN trained with 33,000 images, we tested our HLS extraction method on three datasets that provided different outdoor scenes (Make3D [15,37], KITTI [38] and our simulated images). Figure 8, Figure 9 and Figure 10 show some qualitative results of our HLS extraction method on the three datasets. We can see that our HLS extraction presents a reliable 3D representation of the observed structure.

## 5. Conclusions

In this work, we have presented a novel method for HLS extraction from a single image. The images processed by our method correspond to outdoors urban scenes. Our method combines four elements: (i) we use a CNN to predict the depth for a single image; (ii) we proposed a depth analysis to removes uncertain depth sections; (iii) we proposed a new graph analysis to segment the depth in semantic orientations (namely: left, right, front, up, down), which enables the grouping of sections whose connectivity could be used to infer HLS; and (iv) the connected sections are used as input in a new CNN architecture, proposed by us, to predict HLS in the shape of cubes and rectangular parallelepipeds.

Regarding the experimental results, we carried out a set of evaluations to assess the classification of the semantic orientations in the single image. These evaluations returned a recall average of 0.879758, i.e., considering the ground-truth, we segmented the 87.9% on average correctly. Furthermore, we have an average precision of 0.908150, i.e., considering our segmented orientation, we segmented 90.8% on average correctly. In addition, the results of all our different segmented orientations have an *F-score* greater than 0.85. We deem these results adequate since the semantic orientations are an essential pre-processing step in our method.

We continued our evaluations to assess the performance of the CNN used to recover the 3D HLS from joint segments detected in the single image. For this, we used a virtual environment based on the simulator Gazebo in order to generate the ground truth for the 3D shapes observed in the single test images. Thus, our four CNN architectures (trained with 33,000 images) obtained an rsm average between 0.35 and 0.36. This shows the stability in the prediction of HLS coordinates. Last, but not least, we carried out a qualitative evaluation of the whole method using three datasets with different outdoor scenes.

The results reported in this work indicate that our methodology is feasible and promising. We envisage that our proposed methodology can be extended to more complex polyhedral structures. In this sense, we should highlight that our goal is not that of fine/dense 3D reconstruction, but to generate a useful abstract representation that can be exploited in different applications ranging from robotics to computer vision. Some concrete application examples we can think of are: occlusion representation during navigation; semantic annotation (buildings, roads); city modelling (using HLS); and augmented and mixed reality applications.

We differentiate our approach from other CNN-based approaches for 3D object pose estimation in the sense that we focus on urban environments, where we aim at interpreting the scene in order to generate abstract representations of buildings. These representations involve the use of HLS in the shape of 3D volumetric primitives such as cubes and rectangular parallelepipeds, whose 3D position and orientation are also estimated w.r.t. to the optical centre of the camera view corresponding to the single image. As shown in some qualitative examples, our method enables the extraction of such parallelepipeds, which can be placed in the same reference coordinate system, thus enabling a high-level 3D representation of what is being observed in a single image.

Finally, we should note that our approach involves a combination of techniques based on CNN architectures with conventional methods for classification (from the machine learning point of view) and rule-based segmentation and data grouping, which in our opinion brings the best of the two worlds to address the difficult problem of a single-image interpretation, which in our case was focused on the task of extracting HLS.

Future work involves further investigation of the improvement of the CNN architectures used in this work. There is also an open question about what steps in our method can be replaced by CNN modules if all the modules or even if the whole problem can be solved using a single CNN architecture. In the mean time, we consider this work and its obtained results to be the first baseline to be overcome. We will also explore the use of more complex parallelepipeds to those used in this work, seeking to find the trade-off between representation and usefulness for the user and/or application.

## Figures and Tables

**Table 1:** Depth estimation using the state-of-the-art and our refined depth. We compare on the Make3D and KITTI datasets.

	Make3D		KITTI	
Method	log_10_	rms	log_10_	rms
Saxena et al. [15]	0.187	-	-	8.734
DCCRF [24]	0.134	**12.60**	-	-
DCNF-FCSP [26]	0.122	14.09	0.092	7.046
**ours**	**0.119**	13.20	**0.086**	**6.805**

**Table 2:** Semantic orientation estimation using the Make3D and KITTI datasets. To provide quantitative results, we use three measures (Recall, Precision and *F-score*).

Orientation	Recall	Precision	F-Score
Down view	0.865789	0.850642	0.858148
Right view	0.831157	0.929074	0.877392
Front view	0.933024	0.878332	0.904852
Left view	0.837613	0.962360	0.895663
Upward view	0.931210	0.920342	0.925744
Average	0.879758	0.908150	0.892359

**Table 3:** We measured the coordinate (*x*,*y*,*z*) vertices of our HLS extraction with the ground-truth. Our CNN training used different simulated element numbers in the comparison. We measured the HLS extraction effectiveness with the root mean squared error (rms).

3D Shape	Training	rms (*x*)	rms (*y*)	rms (*z*)	Average rms
**Cube**	500	0.307791	0.974300	2.344010	**1.208700**
	5000	0.168358	0.858216	1.155927	**0.727500**
	10,000	0.170676	0.492700	0.848051	**0.503809**
	17,500	0.176459	0.329077	0.667617	**0.391051**
	25,000	0.139411	0.332585	0.655419	**0.375805**
	33,000	0.135793	0.333141	0.589185	**0.352706**
**Half cube**	500	0.293828	0.987950	2.265533	**1.182437**
	5000	0.172247	0.799870	1.161913	**0.711343**
	10,000	0.171351	0.510368	0.835438	**0.505719**
	17,500	0.166451	0.315625	0.656397	**0.379491**
	25,000	0.145339	0.316189	0.663797	**0.375108**
	33,000	0.136711	0.305686	0.619623	**0.354006**
**Horizontal rectangle**	500	0.331915	1.012385	2.452832	**1.265710**
	5000	0.186942	0.766469	1.115302	**0.689571**
	10,000	0.199112	0.663864	0.765380	**0.542785**
	17,500	0.165131	0.677991	0.704600	**0.515907**
	25,000	0.164179	0.337327	0.645978	**0.382494**
	33,000	0.154344	0.299278	0.631273	**0.361631**
**Vertical rectangle**	500	0.261852	0.963921	2.263405	**1.163059**
	5000	0.176730	0.813496	1.190372	**0.726866**
	10,000	0.180218	0.743857	0.637044	**0.520373**
	17,500	0.156088	0.701990	0.693192	**0.517090**
	25,000	0.158378	0.321481	0.721955	**0.400604**
	33,000	0.168115	0.303499	0.613946	**0.361853**
